# The Interplay between Finasteride-Induced Androgen Imbalance, Endoplasmic Reticulum Stress, Oxidative Stress, and Liver Disorders in Paternal and Filial Generation

**DOI:** 10.3390/biomedicines10112725

**Published:** 2022-10-27

**Authors:** Sylwia Rzeszotek, Agnieszka Kolasa, Anna Pilutin, Kamila Misiakiewicz-Has, Katarzyna Sielatycka, Barbara Wiszniewska

**Affiliations:** 1 Department of Histology and Embryology, Pomeranian Medical University in Szczecin, 70-111 Szczecin, Poland; 2 Institute of Biology, Faculty of Exact and Natural Sciences, University of Szczecin, 71-415 Szczecin, Poland

**Keywords:** finasteride, androgen imbalance, androgen receptor, endoplasmic reticulum stress, oxidative stress, liver, filial generation

## Abstract

Finasteride (Fin) causes androgen imbalance by inhibiting the conversion of testosterone (T) to its more active metabolite, dihydrotestosterone (DHT). Androgen receptors (AR) are present (e.g., in hepatocytes), which have well-developed endoplasmic reticulum (ERet). Cellular protein quality control is carried out by ERet in two paths: (i) unfolded protein response (UPR) and/or (ii) endoplasmic reticulum associated degradation (ERAD). ERet under continuous stress can generate changes in the UPR and can direct the cell on the pathway of life or death. It has been demonstrated that genes involved in ERet stress are among the genes controlled by androgens in some tissues. Oxidative stress is also one of the factors affecting the functions of ERet and androgens are one of the regulators of antioxidant enzyme activity. In this paper, we discuss/analyze a possible relationship between androgen imbalance in paternal generation with ERet stress and liver disorders in both paternal and filial generation. In our rat model, hyperglycemia and subsequent higher accumulation of hepatic glycogen were observed in all filial generation obtained from females fertilized by Fin-treated males (F1:Fin). Importantly, genes encoding enzymes involved in glucose and glycogen metabolism have been previously recognized among UPR targets.

## 1. Basic Components of the Endoplasmic Reticulum Stress Machinery

The endoplasmic reticulum (ERet) is responsible for protein synthesis, calcium storage and regulation of its metabolism, lipid synthesis and storage, and glucose metabolism. This organelle coordinates the dynamics of metabolic responses and energy fluctuations to finally decide the cell’s destiny. The functions of ERet are sensitive and dependent on many variables (e.g., accumulation of misfolded proteins), which may be caused by genetic mutations in the cancer cells or local cell proliferation, leading to a temporary decrease in local energy (glucose) or oxygen levels. Other ERet stimulants include a large group of factors such as multiple ERet stressor molecules, inappropriate temperature, reactive oxygen species (ROS), and many others. In response to stressors, ERet activates mechanisms that adapt the organelle and cell to a stressful situation [1,2,3]. The three main signaling pathways—IRE1 (inositol-requiring transmembrane kinase/endoribonuclease 1α), PERK (protein kinase R-like endoplasmic reticulum kinase), and ATF6 (activating transcription factor 6)—are kept inactive by the heat shock protein chaperone, GRP78 (78-kDa glucose-regulated protein also known as BiP, binding immunoglobulin protein). GRP78 is primarily located in the ERet lumen with a subfraction detected as a transmembrane protein [4,5,6]. Amino ends -NH_2_ of IRE-1, PERK, and ATF6 are located in the lumen of ERet while carboxyl ends -COOH are located in cytosol [7,8].

IRE1α is the most conserved of the three UPR (unfolded protein response) pathways and is expressed in the liver [9].

Triggering the signaling cascade under stress starts a powerful arsenal of signaling molecules together called the UPR [10,11,12,13].

Recent data indicate that the activation of these receptors is more complex than only activation by GRP78 dissociation. For example, yeast research demonstrated that self-association and GRP78 dissociation are not sufficient for the activation of the ER stress sensor Ire1 [14]. There are some data indicating that unfolded proteins can also bind to the luminal domains of PERK [15] and IRE1 [16] and can trigger their oligomerization.

As oligomer IRE1 autophosphorylates and activates the endoribonuclease domain, which performs unconventional splicing of X-box-binding protein 1 (XBP1). Spliced XBP1 (sXBP1) is a potent transcription activator of endoplasmic reticulum associated degradation (ERAD) components [17]. Activated PERK initiates the translation of i.a. activating transcription factor 4 (ATF4), which in response controls the cellular response and UPR pathway [18]. When ERet experiences stress, ATF6 is translocated to the Golgi, where it is cleaved and later transported to the nucleus, where it controls the expression of chaperones (e.g., GRP78), or together with XBP1s, increase the expression of ERAD components [17,19].

## 2. Meaning of Unfolded Protein Response

The ERet is the site for the synthesis, folding, modification, and secretion of proteins. The most common causes of ERet stress are impaired protein glycosylation or disulfide bond formation and the overexpression of misfolding protein prepared for secretion [20], which then activate a cascade of signals (e.g., some genes, enzymes, and ubiquitination processes—UPR). The role of UPR is to protect the organism from misfolded proteins and restore homeostatic conditions. ERet stress is connected with an excess of ERet-resident chaperone proteins by the load of misfolded proteins [21]. Cellular survival depends on (i) restoring homeostasis (e.g., via blocking protein translation); (ii) leading to ubiquitination and degradation of misfolded proteins; and (iii) proper reaction of chaperones. Sometimes, chronic stress can also lead a cell to apoptosis [22]. It has been demonstrated that when stress occurs, UPR acts via three receptors; (i) PERK; (ii) ATF6; an (iii) IRE1. These receptors are inactive in non-stressed cells since they are connected with chaperon protein GRP78/BiP (78-kDa glucose-regulated protein, binding immunoglobulin protein). In stress responses, receptors are dissociating and become activated, leading to the beginning of ERAD. However, the exact role of GRP78 is still being discussed [23]. ERet stress is correlated with the lysosomal pathway of autophagy. It was also demonstrated that entering the UPR pathway can stimulate or inhibit autophagy [24]. Growing data indicate that the long-lasting activation of UPR plays an important role in cancer of the liver, hyperglycemia, obesity, hepatic disorders, or cardiovascular diseases [25]. Many previous studies have demonstrated that ERet, as the side of lipid metabolism, under the prolonged stress condition can promote liver steatosis, non-alcoholic fatty liver disease (NAFLD), and many others [26].

## 3. Finasteride, Testosterone and Androgen Receptor

Testosterone (T), the main male sex hormone, is synthesized from cholesterol, mostly by Leydig cells and, in small amounts in the form of pro-androgens (e.g., dehydroepiandrosterone, DHEA), which require conversion to T by Sertoli cells in seminiferous tubules and the cortex of the adrenal gland [27]. It is also produced in females by the ovaries (including postmenopausal ovaries) as well as the zona reticularis of adrenal glands in the form of pro-androgens [28]. There are some factors affecting changes in the level of T i.a. age, annual rhythm, weight, diet, hormonal balance (e.g., LH concentration), health condition (tumors) [29], environmental endocrine disruptors (EED) [30,31], and some therapies (e.g., finasteride treatment) [32]. Finasteride is the steroid inhibitor of 5α-reductase, which can alter the androgenic homeostasis of the organism by inhibiting the reduction of T to its most active metabolite, 5α-dihydrotestosterone (DHT) [33]. The effects of androgens can be genomic when T or DHT binds to the androgen receptor (AR), or non-genomic via non-DNA binding-dependent actions (e.g., changes in nitric oxide release, local Ca2+ concentration, vascular smooth muscle cell apoptosis, or ROS generation) [34].

Finasteride (Fin) is usually used in the treatment of androgenetic alopecia and benign prostatic hyperplasia [35]. Fin alters androgenic homeostasis and thus causes hormonal imbalance in the body and can therefore be considered as an endocrine disruptor (ED) [36,37]. Systematic reviews predict a potent role of Fin therapies in both males and females, and indicate some side effects (e.g., lower libido and some sexual dysfunctions [38], sweating, changes in the breast or nipples, runny or stuffy noses, some behavioral changes, sleepiness and even depression) [39]. One of the studies that evaluated the effects of Fin treatment was a Prostate Cancer Prevention Trial, which showed that the 7-year risk of prostate cancer was lower by 24.8% in men with PSA (prostate specific aSntigen) at 3.0 ng/mL or less compared to the control. Unfortunately, the high-grade tumor risk (37%) was observed to exceed the placebo (22%) [40]. Thus, it is likely that Fin has two faces and can exert different or even opposite biological effects. Fin may also act as a ligand of the androgen receptor (AR) and change AR expression in different tissues. Research conducted on a cell line derived from Caucasian human prostate carcinoma (LNCap), in a model of androgen-dependent prostate cancer, revealed that the exposure of cells to Fin for 1–6 months increased AR expression [41]. A study reporting symptoms consistent with post-finasteride syndrome (PFS) was conducted on samples of penile skin from 26 men with a history of using 5-alpha-reductase inhibitors, and showed significantly higher AR expression compared to the controls [42]. It has also been demonstrated that AR and the gene expression of UPR are correlated in prostate cancer. Interestingly, AR can bind to UPR and UPR-associated genes [43]. Interestingly, Li et al. [44] observed that AR overexpression increased ERet stress-responsive gene expression in human hepatocytes, and that AR-knockdown led to the downregulation of ERet stress mediators.

## 4. Androgens, Endoplasmic Reticulum Stress, and Liver

**Figure 1:**
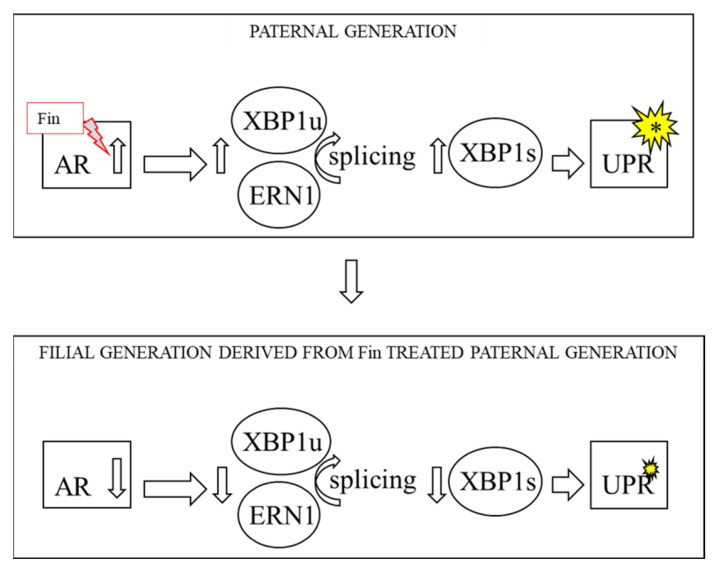
Androgen receptors can modulate the splicing of XBP1s via the ERN1 pathway, which in response activates UPR signaling. We propose that Fin treatment in the paternal generation can also change the AR receptor balance in mature filial generation. (**Upper panel**) Effects of Fin in the paternal generation. (**Lower panel**) Effects of treatment of the paternal generation with Fin in mature filial generation. AR—androgen receptor; Fin—finasteride; XBP1s—X-box binding protein 1 (spliced), XBP1u—X-box binding protein 1 (unspliced), ERN1—endoplasmic reticulum-to-nucleus signaling 1, UPR—unfolded protein response.

In the liver, ARs are expressed in the nuclei of 50–90% of hepatocytes [45]. Hepatocytes, the major population of liver cells, are very active in the synthesis of huge amounts of proteins and lipids, their release, and export [46]. Therefore, they have well-developed cellular organelles, especially rough ERet (rERet) and smooth ERet (sERet). Equal amounts of sERet and rERet are present only in pericentral hepatocytes. Disturbed protein formation in ERet leads to ERet stress and activates a cascade of signals—a mechanism called UPR [21]. Cellular protein quality control is carried out by ERet in two paths: (i) UPR and/or (ii) ERAD. While the ERAD is responsible for proteosomal degradation of misfolded proteins in ERet, the UPR is activated in the case of the accumulation of misfolded protein. Additionally, both pathways have some crosstalk, based on the IRE1α signaling branch [47]. It was also demonstrated that both UPR and ERAD are regulated by androgens [48,49]. In light of these reports, we want to theoretically consider that Fin administration and the associated androgen imbalance can affect the functions of the liver and hepatic ERet. ERet under continuous stress can generate changes in UPR and can direct the cell on the pathway of life or death. ERet stress is also associated with autophagy—a mechanism responsible for inter alia keeping cellular homeostasis [50]. Interesting observations were noted in the liver homogenates obtained from the adult rats (PND90, 90 postnatal days of life), which were the offspring (F1:Fin) born from females fertilized by the Fin-treated male rats (F0:Fin). In a series of tests, it was demonstrated that the expression of ARs on the mRNA level was statistically reduced in adult F1:Fin rats (Figure 1), but not in the younger offspring (7PND, 14 PND, 21 PND, 28 PND). Interestingly, in the F1:Fin group of rats, there was a statistically significant negative correlation between mRNA of T/DHT and AR mRNA level. Importantly, we did not find any such correlations between the level of T and AR transcripts as well as DHT and AR in the offspring born from females fertilized by the control male rats (F1:Control). Our data are quite surprising, however, it should be noted that it relates to offspring born from females fertilized by Fin-treated males, the next generation after the paternal Fin therapy [45]. It is worth noticing that Howell et al. [35] demonstrated that not only AR expression was changed in the penile skin of the 5-alpha-reductase inhibitor treated patients, but also that 1446 genes were significantly overexpressed and 2318 genes were significantly downregulated.

Recently, we also showed that the paternal finasteride treatment can influence the testicular transcriptome profile of male offspring (F1:Fin). We found that in a total of 22,344 genes of the immature rats F1:Fin 14PND, 100 genes were upregulated, 107 downregulated and 22,137 unchanged compared to the F1:Control 14PND. In the sexually mature individuals F1:Fin 90PND, 49 genes were upregulated, 97 downregulated, and 22,198 unchanged compared to the F1:Control 90PND. Finally, during physiological aging, both in the control (F1:Control) and experimental (F1:Fin) groups, a very similar number of genes were upregulated (759 and 724, respectively), downregulated (194 and 154, respectively), or unchanged (21,391 and 21,466, respectively) [51]. It can therefore be assumed that some of the effects induced by paternal finasteride may be transgenerational.

The most attention has been given to genes regulated by androgens in prostate research. In 2002, Segawa et al. [52] documented, using molecular studies and bioinformatics analyses, that genes involved in ERet stress are among the genes regulated by androgens in prostate cancer tissue. The androgen-dependent genes include, among others: (i) endoplasmic-reticulum-associated chaperone ORP150 (oxygen-regulated protein) with anti-apoptotic properties [53]; (ii) protein catalyzing the formation of disulfide bonds PDI (protein disulfide isomerase) from the ERet oxidoreductase family [54]; (iii) localized in ERet Herpud1 protein (homocysteine inducible ERet protein with ubiquitin like domain 1) involved in ERAD and controlled by the stress transcription factor Nrf1 (nuclear respiratory factor 1) and N-myc downstream regulated gene-1 (Ndrg1) [55,56]. Additionally, Ndrg1 is known as a metastasis suppressor and can regulate the main elements of the ERet stress response by (i) increasing the expression of chaperone proteins (BiP), calreticulin, and calnexin; (ii) PERK suppression; (iii) inhibition of the IRE 1α pathway; and (iv) cleavage of ATF 6. An earlier report also showed marked upregulation of NDRG1 in response to androgen in human prostate cancer cellular model LNCaP [57]. It was also observed that the expression of androgen-controlled Herpud1 and Ndrg1 genes was lower in prostate tumor samples from patients and additionally correlated with the tumor grade [52].

In the model describing the protective effect of polyunsaturated fatty acid derivatives on liver metabolism in a high-fat diet and the associated activation of ERet stress pathways, it was observed that ORP150 activation weakened the lipid-induced ERet stress, thus contributing to the improvement in the fat metabolism parameters. These results were confirmed both in vitro on human hepatoma cells, HepG2 cells, and in male mice [58].

However, in the case of such complex mechanisms with a delicate balance such as ERet stress, no simple solutions should be expected. Divergent control of the UPR pathway has been demonstrated in a model of prostate cancer cell lines and using human prostate cancer xenograft CWR22 in animals. The androgen-stimulated LNCap cell line was characterized by an increase in the expression level of IRE1α (inositol-requiring enzyme 1) and XBP1-spliced. Importantly, the levels of other genes involved in the XBP-1 pathway also increased. On the other hand, the expression of elements of the signaling pathway branch associated with PERK under the influence of androgens was attenuated. Additionally, despite the lack of differences in the level of ATF 4 expression on the mRNA level, the protein level increased. In the next step of the ATF 4 downstream, CHOP levels also decreased at the mRNA level, but increased at the protein level. Interestingly, this work also showed that AR has the ability to bind to regulatory sites in genes involved in the UPR [59].

## 5. Oxidative Stress and Endoplasmic Reticulum Stress

Mitochondria and ERet are in contact through the mitochondria-associated endoplasmic reticulum membranes (MAMs) [60]. MAMs are involved in autophagy, Ca^2+^ transport, and lipid metabolism and their alteration is associated with many diseases and hemostasis dysregulation including oxidative stress and autophagy dysregulation [61,62,63]. Oxidative stress is one of the factors affecting the functions of ERet. The level of antioxidant expression (e.g., superoxide dismutase, SOD, or catalase, CAT) is different in the tissues of the reproductive system derived from young and older animals. Androgens are one of the regulators of antioxidant enzymes. In the model of endothelial cells stimulated with T and DHT, an increased level of reactive oxygen species was observed simultaneously with increased SOD and decreased CAT, and those effects were AR dependent [64]. In a cellular model of prostate cancer, androgen induced the production of ROS. It has been hypothesized that when androgens stimulate ROS production, this can cause adaptive changes to oxidative stress and as a final result, lead to the development of treatment resistance (e.g., radiation resistance) [65,66]. Interestingly, in the nervous tissue, T depletion resulted in increased damage through the oxidative stress pathway. Following a gonadectomy of male rats, in the hippocampus isolated from rats, lower levels of antioxidant enzymes (SOD, CAT, glutathione, GSH), increased lipid peroxidation (LOP), and increased apoptotic rate were observed [67,68]. Importantly, ERet stress generates ROS in mitochondria, which also generates feedback and further promotes ERet stress. One of the reasons for oxidative stress in mitochondria is glucose overload and oxidative phosphorylation. Additionally, reactive oxygen species can also induce changes in glucose and lipid metabolism contributes to the accumulation of cholesterol in the liver during aging [69,70,71,72,73].

## 6. Relationship between Androgen Imbalance and Endoplasmic Reticulum Stress

The work of Soni et al. [74] demonstrated that Fin treatment promoted the main proteins related to ERet stress: GRP-78, phoshoprylated-IRE1, and phosphorylated JNK54 (C-Jun N-terminal kinase), which were elevated compared to the control. There are some data indicating a bidirectional correlation between oxidative stress and ERet stress [75]. Glyphosate, a widely used herbicide, is a known endocrine disruptor [76] that decreases the serum T levels by reducing the level of T synthesis and CYP17A1 encoding cytochrome P450, enzymes involved in steroidogenesis. Subsequent increased levels of ERet chaperone protein GRP78/BiP and the phosphorylation of PERK (enzyme activated in response to ERet stress) and eIF2α (eukaryotic initiation factor 2, required for most forms of eukaryotic translation initiation) were demonstrated after glyphosate stimulation of the Leydig cell line [77]. ERet stress and subsequent unfolded protein response are known factors affecting the glucose metabolism in different cells (e.g., neurons or myoblasts via promotion of glycogen formation) [78]. Genes encoding enzymes involved in glucose and glycogen metabolism have previously been recognized among UPR targets. ERet stress orchestrates glycogen synthase kinase 3β (GSK3β), one of the regulators of glycogen synthesis, but also orchestrates the cellular processes. Additionally, derivatives of valproic acids can protect from an excess of lipid accumulation by inhibiting GSK3 and the inhibition of ERet stress decreases GSK3β activity [79]. The inhibition of expression in the liver of metabolic enzyme CYP4A (cytochrome P450 4A) reduced the levels of ERet stress, insulin resistance, and apoptosis in the livers of diabetic mice (db/db). Inversely, the induction of CYP4A accelerated ERet stress, insulin resistance, and apoptosis in the livers of db/db mice [80]. Hyperglycemia is a state observed in all ages of the filial generation obtained from females fertilized by Fin-treated males (F1:Fin). GLUT2 (glucose transporter-2) mediates glucose transport in the liver and pancreas. F1:Fin have a higher accumulation of hepatic glycogen as a result of hyperglycemia, a lower expression of GLUT-2, IR (insulin receptor), and AR in the liver, which are symptoms of liver steatosis as well as higher body weight [81]. Studies conducted by Maseroli et al. [82] revealed that T treatment was connected with visceral fat reduction in men and an improvement in symptoms of NAFLD. AR knockout (ARKO) male mice developed late-onset obesity, and liver-specific ARKO male mice were characterized by increased insulin resistance and steatosis, with decreased β-oxidation, upon being fed a high-fat diet (HFD). These data are clinically relevant since increased IR and impaired glucose tolerance have been observed in men with a low testosterone level [83]. A population-based study [84] also demonstrated that a low serum testosterone level correlated with a higher risk of liver steatosis. This was confirmed additionally in a murine model with 5α-reductase knock-out [85]. Liver lipid metabolism can be affected by the testosterone level as well as AR, and these effects can be independent. Low levels of testosterone predict the later development of type 2 diabetes (T2D) or metabolic syndrome (MetS) with increased levels of low-density lipoprotein (LDL) cholesterol and triglycerides, and with decreased HDL levels. Individuals with HCC (hepatocellular carcinoma) express augmented levels of ARs in their tumor tissue and in the surrounding liver [86].

Interesting observations were made in the C2C12 (mouse myoblast cell line), Hep G2, and 3T3-F442A (mouse embryonic fibroblasts) cell lines where ERet stress for ≥12 h inhibits insulin-stimulated AKT phosphorylation. Additionally, many studies have reported that ERet stress lasting for a minimum of 12 h can cause insulin resistance in the cellular model of skeletal muscles [87] or excessive ER stress desensitized insulin receptor signaling in the model of human embryonic kidney cells and ATF6 protected the insulin pathway from ER stress-induced desensitization. However, ATF6 protection against insulin resistance was not directly linked with its function in ERet stress [88]. It seems that ERet stress is a condition promoting hepatic insulin resistance and steatosis. It is worth noting that Kruppel-like factor 15 (KLF15) was identified as an important mediator of ERet stress. The authors point out that KLF15 deficiency (in murine model) results in the uncoupling of hepatic ER stress and insulin resistance [89]. It was also demonstrated that the overexpression of KLF15 increased the testosterone formation at least 3-fold in cultured H295R cells (human adrenal corticocarcinoma). Additionally, the activity of type 5 17beta-HSD, one of the seven types of 17beta-hydroxysteroid dehydrogenase (17beta-HSD), which catalyzes the transformation of 4-androstenedione into testosterone (T), was increased [90]. KLF15 is another potential link between the androgens [91], ERet stress, and fat stores by insulin in humans.

## 7. Androgens, Endoplasmic Reticulum Stress, and Liver Disorders

The effects of ERet stress on AR expression and intracellular signaling in the liver are unclear. Li et al. [44] demonstrated that in the luminal androgen receptor triple-negative breast cancer (LAR TNBC) and prostate cancer (PCa) cell lines, stimulation with factors triggering the stress of the ERet was associated with a decrease in the level of AR expression at the transcriptional and protein levels. This effect was dependent on the PERK/ATF4 pathway. This paper demonstrated that AR can be an element of the ERet stress pathway. Elevated AR was observed in hepatocellular carcinoma; however, its role is complex. The AR knock-out in induced the hepatocellular carcinoma cellular model showed delayed initiation of cancerogenesis, while on the other hand, tumor growth and metastasis were intensified later. In the patient samples, a lower AR correlated with higher metastatic risk and poorer survival in liver cancers. The authors also paid attention to the specific type of peritumoral vascularization and the expression of Rac (plasma membrane-associated small GTPase) and angiopoietin [92].

**Figure 2:**
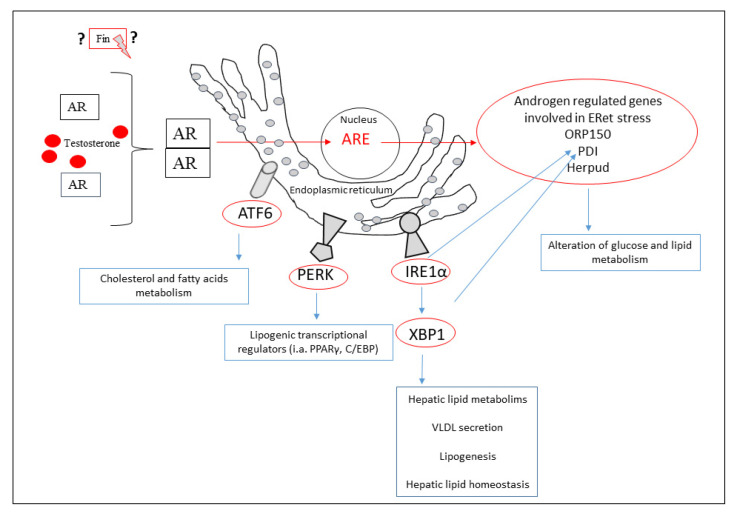
The influence of the Eret stress pathway components on the metabolic processes of lipids in the liver. In the red circles, pathway elements have been marked, the expression of which has been described in the literature, as altered by androgens or their disturbed metabolism. Testosterone binds to androgen receptors (AR), which dimerize and translocate to the nucleus, where they can attach to androgen response elements (AREs) and control the expression of androgen regulated genes (ORP150—oxygen-regulated protein 150, PDI—protein disulfide isomerase, Herpud—homocysteine inducible ER protein with ubiquitin like domain 1). Androgens also affect ATF6, protein kinase R-like endoplasmic reticulum kinase (PERK), inositol-requiring transmembrane kinase/endoribonuclease 1α (IRE), and X-box binding protein 1 (XBP1). Fiansteride (Fin) may be an additional element modulating ERet stress by influencing the level of androgens. Note: XBP1 is characterized by variable expression depending on the severity of symptoms in some diseases.

Little is yet known about the effects of androgen metabolism on endoplasmic reticulum stress markers in hepatic tissue. The major involved components are summarized in Figure 2.

Neoplastic tissue has been examined more intensively (e.g., in the hepatocellular carcinoma model). It is reported that DHT via RNA-dependent protein kinase/eukaryotic initiation factor-2α/GADD153 can affect ERet stress (enhanced) [93]. Grp78/BiP are encoded by androgen-dependent genes that are expressed both in the prostate and in the liver and a positive correlation was observed between the expression level of AR and GRP78 at the mRNA level, especially at the earlier stages of HCC differentiation. Additionally, it was noted that AR overexpression increased the expression of genes involved in ERet stress [86].

NAFL is a major problem today, increasing the risk of death, type 2 diabetes, and cardiovascular disease. Currently, the most widely discussed cause of the disease is the so-called “multiple-hits hypothesis”. The first step leads to steatosis and the following hits cause non-alcoholic steatohepatitis (NASH) [56]. One of the hits are mitochondrial dysfunction, activation of Kupffer cells, oxidative stress, or ERet stress. With the development of chronic liver disease (CLD) of various etiologies, changes in the hormonal balance including the metabolism of sex hormones, cortisol, and insulin have been described [94]. It is known that the incidence of certain liver diseases such as steatosis, hepatitis, liver cancer, and cirrhosis is related to gender. The action of androgens reduces the symptoms of steatosis in rat models, and a known effect of testosterone therapy in men is a decrease in visceral fat. In a murine model (orchiectomized) of non-alcoholic steatohepatitis induced by a high fat diet (NAFLD), it was observed that DHT is associated with reduced lipid accumulation and cholesterol synthesis in cells closest to the triad portal [95]. The problem is that the reported observations are not completely consistent. Androgens have also been reported to promote NAFLD in some cases [96].

Numerous data indicate that ERet stress and UPR signaling pathways participate in many disorders of the liver (e.g., NAFLD, alcoholic liver disease (ALD), alpha-1 antitrypsin deficiency (AATD), cholestatic liver disease, drug-induced liver injury, ischemia/reperfusion (I/R) injury, viral hepatitis, and HCC) [26]. Recent studies show that the components of the UPR pathway can also regulate hepatic steatosis and the cellular response to lipotoxic stress independently of misfolded protein accumulation (so called lipotoxic and proteotoxic stress) [97]. Our data indicate that steatosis was present in the adult offspring of Fin-treated paternal generation, while in the control group, there were no signs of steatosis [81]. DHT, similarly to T, according to Shen et al. [43], is crucial for glucose homeostasis by regulating hepatic glucose output, and T deprivation due to castration increases the hepatic glucose output, induces hyperglycemia, and develops symptoms seen in T2D and MetS. We showed hyperglycemia in the F1:Fin rats (the offspring derived from finasteride-treated parental rats). However, the exact cause of elevated glycogen content in the liver of the F1:Fin rats (lack of degradation or increased synthesis) has yet to be evaluated. It was also observed that patients who received AR-targeted therapies had significantly changed XBP1 and the active form of XBP1 (XBP1s), one of the key factors of UPR [48]. Hence, it seems reasonable to check the effects of Fin on the UPR pathway. In particular, the pattern of AR expression in the liver is associated with disorders such as visceral obesity, leptin, and insulin resistance, and lipid metabolism disorders [98]. In the future, we would like to address the question of whether steatosis and hyperglycemia in the offspring of finasteride-treated paternal rats is related to UPR.

## 8. Autophagy, Unfolded Protein Response, and Liver

Autophagy is an intracellular degradative pathway that targets the cytosolic components to lysosomes to be degraded for the purposes of maintaining cellular homeostasis [99]. As it is a very complex process, the criteria for assessing autophagy are continuously being updated by scientists [100]. In the liver, autophagy has a role in maintaining homeostasis via controlling the energy balance and removing damaged structures (e.g., proteins or lipid droplets) [101]. Additionally, autophagic processes play a role in regulating and preserving liver homeostasis [102]. Recent data have indicated that UPR may induce autophagy, which is able to reduce hepatic UPR [103,104]. However, the mechanism of this balance remains poorly understood. We would like to add one more component to this mechanism, which means androgens, since it was demonstrated that in prostate cancer, androgens can change the expressions of genes involved in autophagy [105]. Interestingly, it was shown that some proteins involved in autophagy (e.g., Grp78/BiP) are up-regulated in some cancers, and are connected with therapeutic failures [106]. Additionally, androgens are able to promote the growth of prostate cancer cells via autophagy [107]. Unfortunately, autophagy is a more complex process that does not simply have a positive or negative effect. Sometimes it can work oppositely and prevent storage disorders, non-alcoholic steatohepatitis, or Wilson’s disease. New data indicate that promising therapy based on autophagy has to have a proper sensitive time window and have biomarkers identified [101]. Moreover, some drugs inducing autophagy have a positive impact on alpha-1 antitrypsin deficiency, a genetic disorder associated with liver disturbances [108]. Growing data indicate that the long-lasting activation of UPR plays an important role in cancer of the liver, hyperglycemia, obesity, hepatic disorders, cardiovascular diseases, and many others [25,26]. Recent studies have shown that components of the UPR pathway can also regulate hepatic steatosis and the cellular response to lipotoxic stress independently of misfolded protein accumulation [97].

## 9. Conclusions

Growing data indicate that androgens can mediate the functions of ERet and UPR in the liver [43,49]. Hyperglycemia and subsequent higher accumulation of hepatic glycogen were observed in rats of filial generation obtained from females fertilized by Fin-treated males (F1:Fin). It seems that ERet stress is a condition promoting hepatic insulin resistance and steatosis. Additionally, androgens are one of the factors controlling components of the oxidative stress response. In the future, it should be considered whether other drugs commonly used may affect the health of offspring by inducing hereditary changes. Attention should be paid to the role of endocrine disruptors (e.g., Fin) in liver disorders. ERet stress connected with UPR may in the future be a therapeutic target to treat liver diseases. Rubinsztein et al. [109] and Allaire et al. [101], in their reviews, believe that one of the challenges in translating autophagy into a clinical trial is a better characteristic of its pathways in liver disorders. We believe that special attention to the characteristics of these pathways should be paid to sex hormones.

## Data Availability

Not applicable.
